# Effect of physiotherapy interventions on attention, hyperactivity, motor and cognitive outcomes in children with attention deficit hyperactivity disorder: a systematic review protocol

**DOI:** 10.3389/frcha.2026.1717704

**Published:** 2026-02-04

**Authors:** Sakshi Desai, Sharath Hullumani, Irshad Qureshi, Rutuja Sawalkar, Aishik Ghosh

**Affiliations:** 1 Department of Paediatrics Physiotherapy, Ravi Nair Physiotherapy College, Datta Meghe Institute of Higher Education and Research (DU), Wardha, India; 2 Department of Neuro Physiotherapy, Ravi Nair Physiotherapy College, Datta Meghe Institute Higher Education and Research (DU), Wardha, India

**Keywords:** attention deficit hyperactivity disorder (ADHD), children, hyperactivity, physiotherapy, systematic review protocol

## Abstract

**Introduction:**

Attention Deficit Hyperactivity Disorder (ADHD) is a prevalent neurodevelopmental disorder among children who attend school, characterized by symptoms of hyperactivity, impulsivity, and inattention. Despite extensive research, the optimal management of attention deficit hyperactivity disorder remains unestablished. The review intends to present evidence-based findings that will address clinical practice and guide future research directions. By integrating the existing findings, it seeks to improve the quality of life as well as their long-term outcomes of children with attention deficit hyperactivity disorder. This systematic review will assess the effectiveness of physiotherapy interventions on attention, hyperactivity, motor, and cognitive outcomes among children with attention deficit hyperactivity disorder.

**Methods:**

The review will synthesize randomized controlled trials (RCTs). To assess relevant English language articles published between Inspection to February 2025, we will perform a systematic search across databases, namely PubMed/MEDLINE, the Cochrane Library, and PEDro, CLARIVATE (Web of Science). The search strategy will use Medical Subject Headings (MeSH) terms as well as relevant words, e.g., “attention deficit hyperactivity disorder”, “pediatrics”, “intervention”, and “physiotherapy”. The risk of bias included in the studies will be evaluated using the Cochrane Risk of Bias Tool (RoB.2). The outcomes regarding attention, hyperactivity, motor function, and cognition for children with ADHD will be synthesized narratively. The variations by study design and characteristics of participants will be further addressed through sensitivity and subgroup analyses.

**Discussion:**

The objective of this systematic review protocol is to assess the effectiveness of physiotherapy interventions on the management of attention deficit hyperactivity disorder symptoms. Analyzing Randomized Controlled Trials systematically will establish evidence-based guidance to improve attention, diminish hyperactivity, and improve motor and cognitive function among children with attention deficit hyperactivity disorder. The outcomes of this review will aid clinicians in well-informed decision-making, optimizing therapeutic approaches, and directing research for the improvement of attention deficit hyperactivity disorder care within the pediatric population.

**Clinical Trial Registration:**

PROSPERO CRD420251037307, 21st April 2025, UTC version 1.0.

## 1 Introduction

Attention deficit hyperactivity disorder (ADHD) is a highly prevalent neurodevelopmental disorder occurring among children of school age (1). In the United States, it is estimated to have a prevalence of 8.9%, whereas it is about 6.5% in China (2, 3). The disorder is mainly characterized by long-term inattention, impulsivity, and hyperactivity, which can adversely affect daily behavior across settings (4). Beyond behavioral symptoms, children with Attention Deficit Hyperactivity Disorder frequently exhibit motor coordination deficits, impaired postural control, sensory processing difficulties, and challenges in self-regulation. These multidimensional impairments provide a strong theoretical basis for physiotherapy as an adjunctive. Attention Deficit Hyperactivity Disorder usually co-occurs with other psychiatric disorders, including anxiety, depression, conduct disorder, and disruptive behavior (5).

The treatment of Attention Deficit Hyperactivity Disorder is usually comprised of medications as well as non-pharmacological interventions (6). Pharmacological management, primarily using psychostimulant medications remains a cornerstone of treatment; however, there is evidence pointing towards side effects of medications, including intolerance, non-responsiveness, as well as dependence potential. Whereas non-pharmacological interventions involve cognitive behavioral training, sensory integration, structured exercise, and aerobic activities have gain prominence in pediatrics Attention Deficit Hyperactivity Disorder management (7).

Physiotherapy interventions have attracted attention as a complementary strategy for alleviating symptoms of Attention Deficit Hyperactivity Disorder, especially regarding motor, cognitive, as well as behavioral outcomes (8). The study pointed towards the effects of physical exercise on catecholaminergic systems, which are similarly addressed by medication for Attention Deficit Hyperactivity Disorder symptoms. Due to this, physical therapy is a useful alternative for alleviating symptoms of Attention Deficit Hyperactivity Disorder (9). A few studies point towards exercise as being beneficial for children suffering from Attention Deficit Hyperactivity Disorder, potentially enhancing attention, self-control, as well as overall behavior (10, 11).

In parallel, recent years have explored growing evidence supporting innovative behavioral and digitally delivered interventions for pediatric mental health conditions. Well-designed non- pharmacological and digital programs have been shown to be feasible, acceptable, and capable of addressing cognitive and emotional needs in children. For instance, Gnazzo et al. (2025) developed and evaluated an internet based cognitive behavioral therapy (iCBT) program for children with anxiety, demonstrating high usability and engagement, thereby highlighting the potential of structured, technology-supported interventions within pediatric mental health care. Such findings reflect a broader shift toward interdisciplinary, child- centered, and non-pharmacological treatment paradigms.

Despite growing interest regarding the use of physiotherapy interventions for Attention Deficit Hyperactivity Disorder, evidence is fragmented, and the effectiveness of various physiotherapy interventions is poorly established. This systematic Review thus seeks to integrate available research on the impacts of physiotherapy interventions on attention, hyperactivity, as well as on motor and cognitive functions among children with Attention Deficit Hyperactivity Disorder. By assessing available literature, this review will provide insights into the potential role of physiotherapy for managing Attention Deficit Hyperactivity Disorder and inform future clinical use.

### 1.1 Research question

What is the effect of physiotherapy interventions on attention, hyperactivity, motor function, and cognitive outcomes in children diagnosed with Attention Deficit Hyperactivity Disorder (ADHD), and how do these intervention parameters, such as each session, the frequency of sessions and the overall duration of the intervention, modulate the effectiveness of physiotherapy intervention in pediatric Attention Deficit Hyperactivity Disorder?

### 1.2 Objectives

This systematic review aims to assess the effects of physiotherapy interventions on attention, hyperactivity, motor, and cognitive outcomes among children with Attention Deficit Hyperactivity Disorder (ADHD). The review is intended to explore and examine different forms of physiotherapy interventions administered to children with Attention Deficit Hyperactivity Disorder. Evaluate the duration, session duration, frequency, and total duration of the intervention to establish its effects on treatment outcomes. Synthesizing evidence for the effectiveness of physiotherapy interventions and clinical implications within the management of Attention Deficit Hyperactivity Disorder using physiotherapy.

#### 1.2.1 Study design

The review will primarily focus on Randomized Controlled Trials (RCTs) that examine the impact of physiotherapy-based interventions on attention, hyperactivity, motor, and cognitive outcomes in the pediatric Attention Deficit Hyperactivity Disorder population.

### 1.3 Type of Population

Included studies must focus on children diagnosed with attention deficit hyperactivity disorder (ADHD) aged between 3 and 17. A qualified healthcare professional should determine a diagnosis using the established criteria in the DSM-5 and ICD-11. Both male and female participants from diverse geographical regions are eligible for consideration.

### 1.4 Type of intervention

This review will encompass studies assessing various interventions for attention deficit hyperactivity disorder (ADHD), to address heterogeneity, interventions will be categorized into the following groups:

Aerobic and structured Exercise Interventions (e.g., Treadmill walking)
Sensorimotor and Sensory Integration-Based Interventions
Mind-body interventions e.g., Yoga)
Neuromodulation-oriented interventions (e.g., Trigeminal nerve stimulation)
Technology- Assisted and Virtual Reality- Based Motor Training (e.g., Exergaming, VR-Assisted movement programs)

### 1.5 Comparison of interest

Included studies must have a comparator group, which may comprise standard care, no treatment, alternative therapeutic interventions, or a placebo.

### 1.6 Outcome measures

#### 1.6.1 The primary outcomes of this review will assess

The level of attention will be evaluated using standardized rating scales: Vanderbilt Attention Deficit Hyperactivity Disorder Diagnostic Parent Rating Scale (VADPRS) and Conners Parent and Teacher Rating Scale (CRS).

Hyperactivity will be assessed using the Conners Rating Scale (CRS) and the Swanson, Nolan, Pelham Teacher and Parent Rating Scale.

#### 1.6.2 The secondary outcomes of this review will assess

Motor function outcomes will be assessed using The Bruininks-Oseretsky Test of Motor Proficiency (BOT) and Weiss Functional Impairment Scale.

Cognitive performance will be evaluated through validated instruments, including the Comprehensive Behavior Rating Scale (CBRS) and Academic Performance Rating Scale.

Changes in the level will be analyzed from baseline to post-intervention and at follow-up assessment.

All outcome measures will be systematically analyzed to determine changes over time and the effectiveness of the intervention.

### 1.7 Exclusion criteria

Studies focusing solely on pharmacological, psychological, behavioral, or educational interventions without any physiotherapy interventions will be excluded. Studies involving participants with comorbid conditions, e.g., Learning disability, autism spectrum disorder, will be excluded. Participants aged above 18 years diagnosed with Attention Deficit Hyperactivity Disorder will be excluded.

### 1.8 Search strategy

A systematic search will be performed across various databases to identify relevant literature examining the effects of physiotherapy interventions on attention, hyperactivity, motor, and cognitive outcomes in children diagnosed with Attention Deficit Hyperactivity Disorder, that is, PubMed (MEDLINE), Web of Science (CLARIVATE), Cochrane Library, and Pedro. The search will be refined by using Medical Subject Headings (MeSH) terms combined with relevant keyword searches using the words “attention deficit hyperactivity disorder”, “pediatrics”, “interventions”, and “physiotherapy”. The search will then be refined using Boolean operators “AND” and “OR”. To capture the most up-to-date and relevant research, secondary searches will be run to locate unpublished studies, as well as to scan for other sources, that is, clinical trial registries, investigator reports, and conference proceedings.

### 1.9 Data management

#### 1.9.1 Study selection

The selection of the study will be carried out in two distinct phases by two independent reviewers, SD and SH. In the first phase, all titles and abstracts retrieved from the database search will be screened to identify potentially relevant studies based on the predefined eligibility criteria. Articles that do not meet the criteria will be excluded at this stage. In the second phase, the full texts of the shortlisted studies will be obtained and assessed in detail by the same reviewers to confirm their suitability for inclusion. Disagreements regarding selection will be resolved through discussion, with a third reviewer (IQ) being contacted if an agreement cannot be reached. The entire process will be documented, and the reasons for exclusion at the full-text stage will be recorded. A PRISMA flow diagram will be prepared to illustrate the number of studies identified, screened, excluded, and finally included in the systematic review.

#### 1.9.2 Data extraction

Two reviewers (SD and SH) will independently extract data using a standardized extraction form, which will be pilot-tested before full application. The extraction form will include information on the characteristics of the studies, participants' demographic data, intervention and comparator details, outcome measurements, and key results. Discrepancies and doubts will be addressed through discussion; a third reviewer (IQ) will be consulted if necessary. In cases where outcome data are missing, incomplete, or unclear, attempts will be made to contact the corresponding authors of the original studies to obtain additional information. If missing data cannot be retrieved, the available data will be analyzed as reported, and the potential impact of missing information will be addressed in the narrative synthesis and sensitivity analyses. Studies with substantial missing data will be interpreted cautiously. Grey literature, including conference abstracts, dissertations, and unpublished trials will not be included in this review due to concerns regarding methodological rigor and incomplete reporting. Only peer-reviewed, full-text randomized controlled trials published in indexed journals will be considered. This decision will be acknowledged as a potential limitation of the review.

#### 1.9.3 Data analysis

To evaluate primary outcomes, such as improvement in attention, behavior, motor, and cognition, the effectiveness of attention deficit hyperactivity disorder treatment will be measured using standardized mean differences (SMD) or relative risks (RR) with their 95% confidence intervals (CI). Because variability between studies is anticipated, data synthesis will mainly use a narrative strategy. The heterogeneity will be systematically explored by organizing findings according to type of physiotherapy intervention, including aerobic and structured exercise programs, sensory integration based therapies, yoga and mind-body interventions, neuromodulation-oriented interventions, virtual reality assisted interventions. Outcomes related to attention, hyperactivity, motor and cognitive domains will be descriptively compared across these intervention categories to identify patterns of effectiveness. Where sufficient data are available, subgroup analyses will further examine variablility in outcomes based on participant characteristics, intervention parameters (session duration, frequency, and total intervention duration), and outcome assessment tools. This will allow exploration of whether specific intervention characteristics influence treatment response. Sensitivity analyses will be conducted to assess the robustness of findings by examining the influence of methodological quality and risk of bias. Studies will be stratified according to Cochrane Risk of Bias (RoB 2) ratings. For enhancing clarity, graphical illustrations will be included with the narrative summary. A meta-analysis is not planned because of variability between studies, although sensitivity analyses will be performed to determine the influence of research design, methodological quality, and risk of bias on reported outcomes. Quantitative synthesis may be considered if a subset of studies demonstrates sufficient homogeneity in intervention type, comparator, outcome measures, and study design. In such cases, pooled analyses using random- effects models will be explored, provided that clinical and statistical assumptions are met.

#### 1.9.4 Quality appraisal

Assessment of the included studies' quality and risk of bias will be conducted using the Cochrane Risk of Bias tool for randomized controlled trials (RCTs) (12). If a sufficient number of studies are available (typically more than 10), a funnel plot will be utilized to assess publication bias. Additionally, Eggers' test or Begg's test will be performed to statistically test for asymmetry on the funnel plot.

## 2 Discussion

### 2.1 Overview

This systematic review protocol aims to examine the effectiveness of physiotherapy interventions for children with Attention Deficit Hyperactivity Disorder (ADHD). It is a neurodevelopmental disorder affecting attention, activity levels, impulse control, play, and social behavior, impacting activities of daily living. This review anticipates that sufficient literature will be available for both quantitative and qualitative analysis, indicating the beneficial effects of physiotherapy interventions in managing symptoms of Attention Deficit Hyperactivity Disorder. This systematic review seeks to identify the most effective physiotherapy interventions in improving attention, reducing hyperactivity, and enhancing motor skills and cognitive outcomes in children diagnosed with Attention Deficit Hyperactivity Disorder. A comparative analysis of various physiotherapy interventions will be conducted to determine their relative impact on these specific outcomes. By synthesizing the available literature on pediatric Attention Deficit Hyperactivity Disorder, this review aims to provide valuable insights into the role of physiotherapy in Attention Deficit Hyperactivity Disorder management, ultimately guiding clinical practices and future research directions.

### 2.2 Principal findings

Despite known data from Randomised Controlled Trials on the beneficial effects of physiotherapy interventions on attention, hyperactivity, motor, and cognitive outcomes, there is a large knowledge gap regarding how effective different forms of physiotherapy interventions are compared to one another. Additionally, it is not yet well established what the optimal parameters for frequency and duration of the physiotherapy sessions, as well as the intensity of these interventions, are for children with Attention Deficit Hyperactivity Disorder.

### 2.3 Strengths and limitations

The purpose of this systematic review is to undertake a comprehensive review of the impacts of physiotherapy interventions on attention, hyperactivity, motor, and cognitive outcomes for children with Attention Deficit Hyperactivity Disorder, highlighting the role of physiotherapy for managing children with Attention Deficit Hyperactivity Disorder. Synthesizing the evidence available from the existing research, the review will establish the most effective physiotherapy intervention for managing symptoms of Attention Deficit Hyperactivity Disorder. The review will also explore the key intervention parameters, such as duration of sessions, frequency of sessions, and overall duration of the intervention, to establish their influence on outcomes from the intervention. The review will aid healthcare professionals' decision-making capability to make evidence-based decisions for developing treatment plans for Attention Deficit Hyperactivity Disorder. The heterogeneity of the included research, through differences across studies concerning their methodology, type of interventions, and outcome measures, might present a difficulty for synthesizing findings and prevent the feasibility of conducting a meta-analysis. It will provide useful clinical insight and guidance to inform practice and research for managing children with Attention Deficit Hyperactivity Disorder for the foreseeable future.
